# State of oral innate immunity and salivary microbiome with allogeneic hematopoietic stem cell transplant

**DOI:** 10.1128/spectrum.00118-26

**Published:** 2026-08-10

**Authors:** G. R. Adami, M. Fine, J. L. Schwartz, P. Wojtowicz, J. Moreira

**Affiliations:** 1 Department of Oral Medicine and Diagnostic Sciences, College of Dentistry, University of Illinois 124033, Chicago, Illinois, USA; 2 Robert H. Lurie Comprehensive Cancer Center of Northwestern University 88966, Chicago, Illinois, USA; 3 Division of Hematology and Oncology, Department of Medicine, Northwestern University Feinberg School of Medicine 12244, Chicago, Illinois, USA; Nova Southeastern University, Fort Lauderdale, Florida, USA

**Keywords:** 16S rRNA gene, saliva, serum, alpha diversity, allogeneic hematological stem cell transplant

## Abstract

Allogeneic hematopoietic stem cell transplantation (allo-HSCT) and the use of chemotherapy and antibiotics cause large changes in the gut and oral microbiomes. In patients, these differences in the oral microbiome may be related to the re-establishment of the innate immunity in the oral cavity. Saliva samples were collected from a pilot group of allo-HSCT patients before transplant and at the time of engraftment, when blood neutrophil count had rebounded. As expected, alpha diversity measures of the salivary bacterial community (bacteriome) were low prior to the beginning of the treatment and even lower at the time of engraftment. To examine the local innate immunity, a DNA epigenetic/methylation-based identification of salivary granulocytes was performed post-HSCT on harvested oral DNA samples. At the time of engraftment, salivary granulocyte levels were elevated in allo-HSCT patients vs the level in healthy controls. Salivary and blood neutrophil concentrations both trended to correlate with the Chao1 alpha diversity of the salivary microbiome in patients at the time of engraftment.There was correlation or trends toward correlation between levels of both hematocrit and platelets in blood and the Chao1 and Shannon alpha diversity of the saliva microbiome. In conclusion, the data analysis suggested that during the time of engraftment of the donor stem cells those patients with lowerst diversity of oral bacteria also had the lowest numbers of oral and blood neutrophils. This may contribute to the known higher risk of health complications in patients with dysbiotic microbiomes post-HSCT.

**IMPORTANCE:**

Like the gut microbiome, the oral microbiome includes a large variety of bacteria. We analyzed changes in oral microbiota after allogeneic hematopoietic stem cell transplantation. We found that patients with abnormally low numbers of different bacterial types after the transplant procedure may have had the most abnormal innate immune cell systems both orally and in the blood. This highlights a possible link between the oral microbiome and the innate immune system during the crucial period of immune reconstitution.

## INTRODUCTION

Allogeneic hematopoietic stem cell transplantation (allo-HSCT) is used to treat blood cancers and blood or immune system disorders by destroying malignant or malfunctioning cells and replacing them with those of a healthy donor (1). Just before the transplant procedure, patients receiving an allo-HSCT require conditioning with the administration of high-dose chemotherapy and/or radiation and antibiotics (2). These immunoablation steps are necessary to destroy the hematopoietic stem cells of the transplant recipient prior to the infusion of a healthy donor’s immune cells. Engraftment takes a median of 25 days after the transplant for allo-HSCT patients (3) and is defined when the patient maintains a threshold for absolute neutrophil and platelet counts for more than three consecutive days without transfusions (4). Because the risk of developing bacterial and fungal infections is high for these immunocompromised allo-HSCT patients, antibiotics are often given to proactively prevent infection, depending on the level of neutropenia and mucosal damage (5). Within 2–4 weeks after transplantation, it has been shown that the innate immune system begins to recover in patients with fewer infections and better survival (6). The adaptive immune system recovers sometime after the innate system (7).

In the short term, allo-HSCT treatment has severe effects on the immune system and the oral and gut microbiome (8–11). Prior to the engraftment of inflammatory immune cells and the recovery of the oral and gut microbiome, patients frequently develop serious complications such as oral mucositis and possibly life-threatening infections (12). Effects on the oral and gut microbiomes can be due to the chemotherapeutic agents used (13), the antibiotics used to treat infections (14), the inflammation seen after treatment, or due to other abnormalities of the immune system (15). With HSCT, large alterations in the gut and oral microbiome have been noted, with decreases in alpha diversity in the gut and oral cavity that add to the low microbial diversity typically found prior to the procedure in these patients (16). Blooms of specific bacterial species can occur in conjunction with mucositis (17). During restoration of the inflammatory immune system in the weeks following cytotoxic conditioning and infusion of new immune cells, the gut and oral microbiome, along with epithelial lining, also return closer to a normal state (18).

Allo-HSCT can result in several long-term outcomes. Approximately 40% of allo-HSCT patients develop graft-vs-host disease (GVHD) after their transplant, making it the most common complication that is potentially fatal. Another possible outcome is transplant rejection. Patients with low survival of donor stem cells may experience relapse and require intervention, such as additional immunosuppressors, donor lymphocyte infusion, or subsequent engraftment. Thus, the development and integration of the donor immune system with that of the recipient are highly important for successful treatment.

Several studies have shown that the state of the oral and gut microbiome at the time of transplantation and the weeks after the transplantation is associated with treatment outcomes, such as levels of GVHD, remaining disease-free, and overall survival (8, 9, 19, 20). For the oral microbiome, there are links between dysbiosis at the time of transplantation and poor outcomes. The same is true for disruption of the oral microbiome induced by allo-HSCT in the weeks after transplant initiation and allo-HSCT outcomes (9). In addition, patients who fail to show recovery of the oral mucosal microbiome by 2–3 months post-allo-HSCT tend to show poor recovery of the immune system and progression-free survival. Similarly, in the gut, several clinical studies have shown that dysbiosis of the gut microbiota and reduced microbial diversity after allo-HSCT are associated with a significantly increased risk of GVHD and mortality (21).

This study intends to analyze the oral microbiome, salivary innate immune cells, and blood immune cells in patients who received an allo-HSCT and postulate the relationship between oral microbes and parts of the rebounding immune system after pre-engraftment conditioning in order to decrease post-transplant complications. It is based on the hypothesis that salivary microbiome, sampled at the time of preconditioning or at the time of engraftment, is associated with the state of the innate immune system in the oral cavity.

## RESULTS

### Patient characteristics

All patients provided complete blood count (CBC) data and pre- and/or post-transplant oral saliva samples, from which oral microbiome data were obtained. Summarized patient characteristics are included in Table 1. Of the 21 patients enrolled, 15 provided pre-transplant saliva samples, and all 21 provided post-transplant samples. The pre-transplant cohort had a mean age of 50.9 years (SD = 13.3), while the post-transplant cohort averaged 50.4 years (SD = 14.4). Both groups were predominantly male (73% pre-transplant and 67% post-transplant) and identified as White (53% pre-transplant and 71% post-transplant). The most common primary diagnosis was acute myelogenous leukemia (AML), followed by non-Hodgkin’s lymphoma (NHL). All grafts were sourced from peripheral blood, and donor sources included haploidentical, matched sibling, and matched unrelated donors in both groups. At the time of 12-month follow-up, survival rates were similar for both groups (80% pre-transplant and 86% post-transplant). Eighteen healthy controls were used for the bacterial taxa analysis, with an average age of 51.2 (SD = 17.2), consisting of 10 males and 8 females, with racial demographics of 15 Caucasian individuals, 2 Asian individuals, and 1 Black individual. Saliva immune cell analysis was performed on eight of these samples.

**TABLE 1:** Patient characteristics of the pre- and post-transplant cohorts^*a*^

Characteristic	Pre-transplant cohort (*n* = 15)	Post-transplant cohort (*n* = 21)
Age (years)	Mean = 50.9 (SD = 13.3)	Mean = 50.4 (SD = 14.4)
Sex	Male: 11 (73%) Female: 4 (27%)	Male: 14 (67%) Female: 7 (33%)
Race	White: 8 (53%) Black: 3 (20%) Asian: 1 (7%) Unknown: 3 (20%)	White: 15 (71%) Black: 3 (14%) Asian: 0 (0%) Unknown: 3 (14%)
Primary diagnosis	AML: 7 (47%) MDS: 2 (13%) NHL: 5 (33%) Other: 1 (7%)	AML: 8 (38%) MDS: 1 (5%) NHL: 7 (33%) Other: 5 (24%)
Conditioning regimen	Bu/Flu: 3 (20%) Bu/Flu/Cy: 1 (7%) Flu/Mel: 1 (7%) Flu/Mel/Thiotepa/Cy: 1 (7%) Other/unspecified: 9 (60%)	Bu/Flu: 3 (14%) Bu/Flu/Cy: 2 (10%) Flu/Mel: 3 (14%) Flu/Mel/Thiotepa/Cy: 1 (5%) Other/unspecified: 12 (57%)
Donor type	Haploidentical: 4 (27%) Matched sibling: 3 (20%) Matched unrelated: 8 (53%)	Haploidentical: 4 (19%) Matched sibling: 5 (24%) Matched unrelated: 12 (57%)
Graft source	Peripheral blood: 15 (100%)	Peripheral blood: 21 (100%)
12-month survival status	Alive: 12 (80%) Deceased: 3 (20%)	Alive: 18 (86%) Deceased: 3 (14%)

^a^ AML, acute myelogenous leukemia; MDS, myelodysplastic syndrome; NHL, non-Hodgkin’s lymphoma; Bu/Flu, busulfan/fludarabine; Bu/Flu/Cy, busulfan/fludarabine/cytoxan; Flu/Mel, fludarabine/melphalan; and Flu/Mel/Thiotepa/Cy, fludarabine/melphalan/thiotepa with post-transplant cyclophosphamide.

Among 11 patients whose saliva was studied for immune cells, the average age was 49.9 years (SD = 13.5); there were eight males and three females, comprising seven White individuals, two Black individuals, and two Asian individuals. Controls used for this analysis had an average age of 58.0 years (SD = 12.3) and included seven males and one female, comprising six Caucasian individuals and two Asian individuals.

### Microbiota dynamics analyses

Saliva samples in this study were taken from allo-HSCT patients before preconditioning (Pre) (1 week prior to the beginning of conditioning and 2 weeks prior to infusion) and/or at the time of engraftment (Eng) (2–4 weeks post-infusion). Additional saliva samples were from healthy controls (Con).

**Fig 1:**
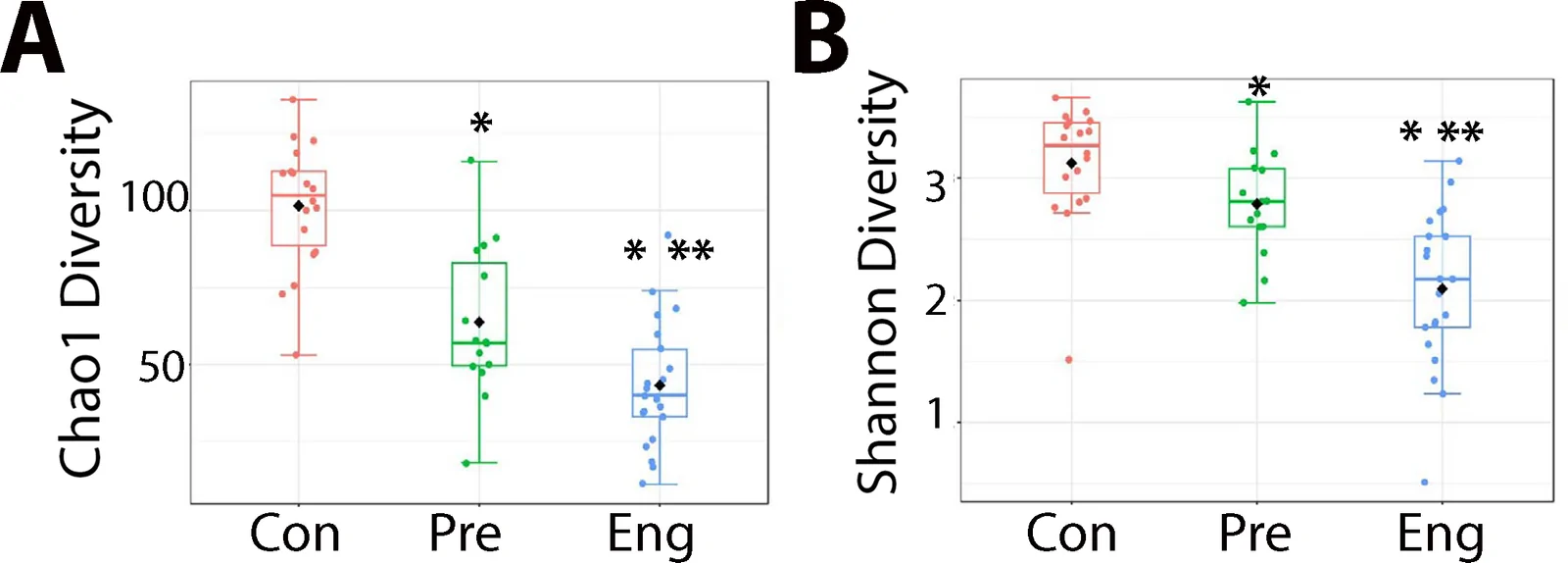
Compositional differences in the salivary microbiome were compared for the three groups: Con, healthy controls; Pre, allo-HSCT patients prior to conditioning; and Eng, allo-HSCT patients at the time of engraftment. (**A**) Comparison of Chao1 alpha diversity. * indicates FDR <0.05 for Pre or Eng samples vs control; ** indicates FDR <0.05 for Pre vs Eng samples. (**B**) Similar to panel **A**, except average Shannon diversity measures were compared.

Alpha diversity, whether measured by the Chao1 index (taxa richness) or the Shannon index (a measure of richness and evenness), was lower in saliva samples from patients than in controls, with the lowest levels observed at the time of engraftment (Fig. 1A and B). Shannon alpha diversity differed significantly between the Pre and Eng groups (*P* = 6.76 × 10^−4^, FDR = 0.00101), the Con and Pre groups (*P* = 0.0460, FDR = 0.0460), and the Con and Eng groups (*P* = 2.44 × 10^−6^, FDR = 7.33 × 10^−6^). Chao1 alpha diversity also differed significantly between the Pre and Eng groups (*P* = 0.021, FDR = 0.021), the Con and Pre groups (*P* = 6.00 × 10^−5^, FDR = 9.0 × 10^−5^), and the Con and Eng groups (*P* = 2.89 × 10^−10^, FDR = 8.66 × 10^−10^).

**Fig 2:**
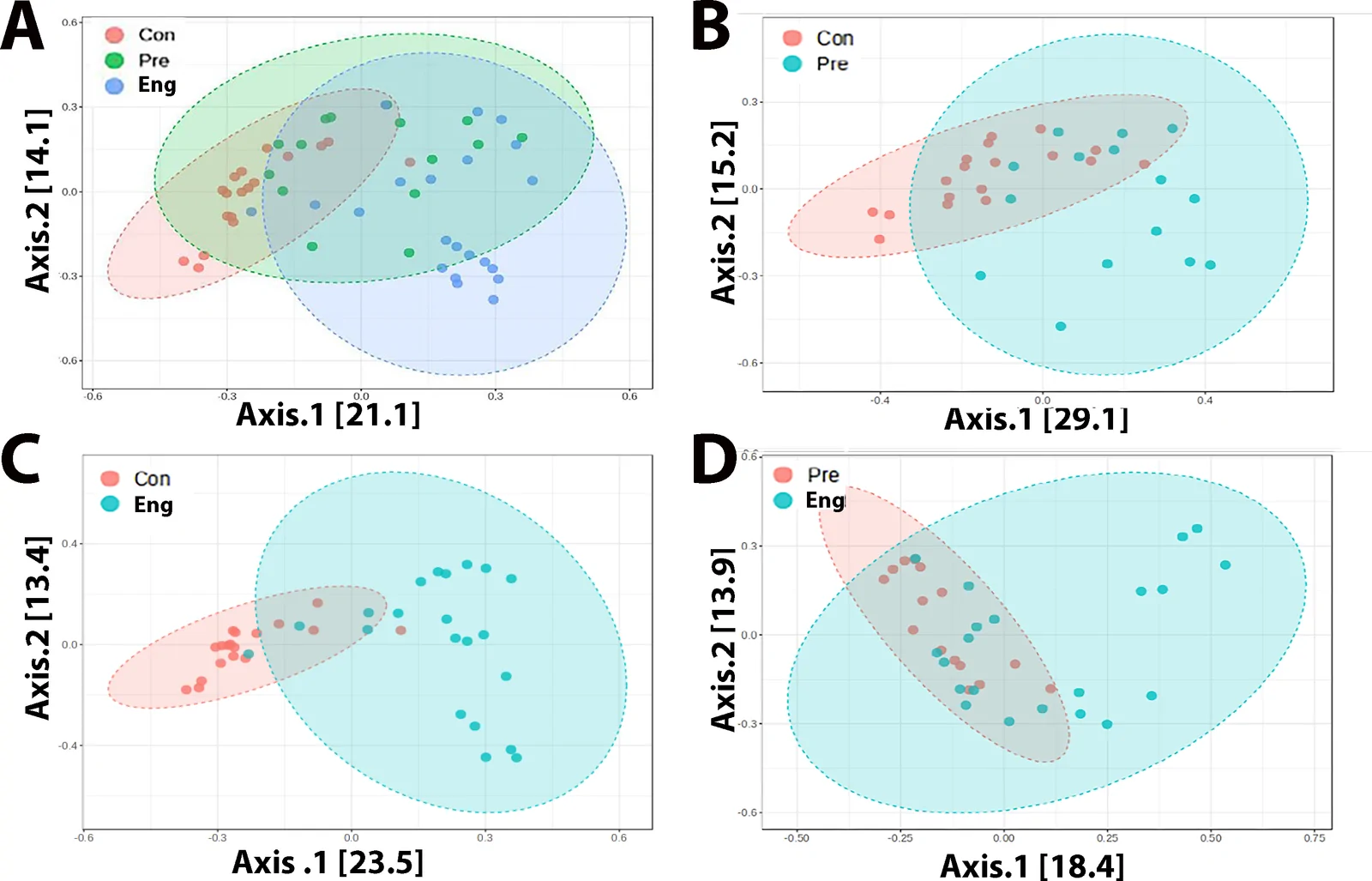
Beta diversity, measured by the Bray-Curtis index, of oral amplicon sequence variants of (**A**) the three groups: Con, healthy controls; Pre, allo-HSCT patients prior to conditioning; and Eng, allo-HSCT patients at the time of engraftment. (**B**) Beta diversity of Con vs Pre. (**C**) Beta diversity of Con vs Eng. (**D**) Beta diversity of Pre vs Eng.

To further understand the level of differences in the oral microbiome between groups, PERMANOVA tests to investigate beta diversity using Bray-Curtis dissimilarity were performed (Fig. 2). When comparing the bacteria from pre-HSCT patients to those of patients at the time of engraftment, significant differences were observed as measured by beta-diversity (*R*^2^ = 0.065737, *P* = 0.006, FDR = 0.006). When comparing control patients to the study patients, significant differences were observed between controls and pre-transplant patients (*R*^2^ = 0.14711, *P* = 0.0010, FDR = 0.003) and between controls and the patients at the time of engraftment (*R*^2^ = 0.17502, *P* = 0.0010, FDR = 0.003).

**Fig 3:**
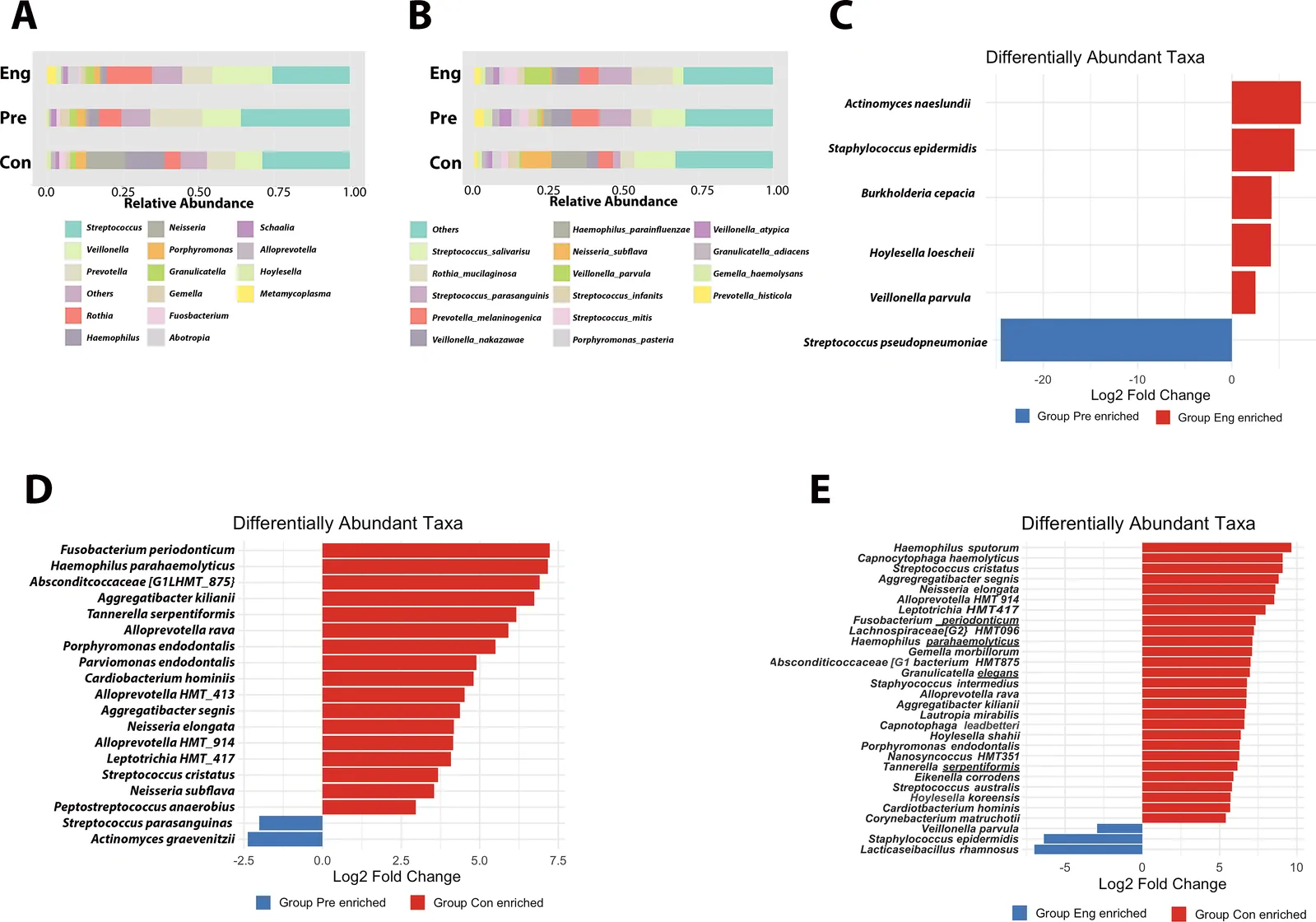
Relative abundances of the oral microbiome across the three groups: Con, healthy controls; Pre, allo-HSCT patients prior to conditioning; and Eng, allo-HSCT patients at the time of engraftment. (**A**) Genus-level abundances of the 15 most abundant taxa, with “Others” representing all other taxa, in the three groups of saliva samples. (**B**) Species-level abundances of the top 15 species, with “Others” representing all remaining species, in the three groups of saliva samples. (**C**) Relative abundance differences in saliva for Pre vs Eng, using DESeq2 at FDR < 0.1. (**D**) Relative abundance differences in saliva for Con vs Pre, using DESeq2 at FDR < 0.1. (**E**) Differentially abundant species in the saliva of Con vs Eng, as identified using DESeq2 at FDR < 0.001.

The bar graphs in Fig. 2A and B show the relative abundance of the 15 major species and genera in each group. Univariate analysis (Fig. 2E) using DESeq2 allowed the identification of taxa differences in the saliva microbiome at the time of Eng vs the Pre samples, which were statistically significant after correction for multiple testing. *Staphylococcus epidermidis, Actinomyces naeslundii, Burkholderia cepacia, Prevotella loescheii,* and *Veillonella parvula* all showed higher relative abundances at the time of engraftment despite the lower overall alpha diversity in these samples vs the Pre saliva samples.

Looking beyond the pre-transplant and time of engraftment comparisons, we noticed additional differences in species- and genus-level abundances when comparing salivary taxa with those of healthy controls. At the species level, 19 taxa were identified as differentially abundant in the saliva of Pre patients compared with healthy controls (FDR < 0.1) (Fig. 3). Forty-nine taxa were differentially abundant in the saliva of the patients at the time of engraftment compared with controls (FDR < 0.1) (Fig. 3D; Table S2). Many of these differences were lower relative levels of specific taxa in patients vs controls (Fig. 3D and E). At the species level, 15 taxa were identified at lower abundance at both time points for the allo-HSCT patients vs the healthy controls (FDR < 0.1). Among the species at lower abundance in controls (Fig. 3D and E; Table S2) vs Pre and Eng samples were several common members of the oral microbiome, including *Streptococcus cristatus* and *Neisseria elongata*.

**Fig 4:**
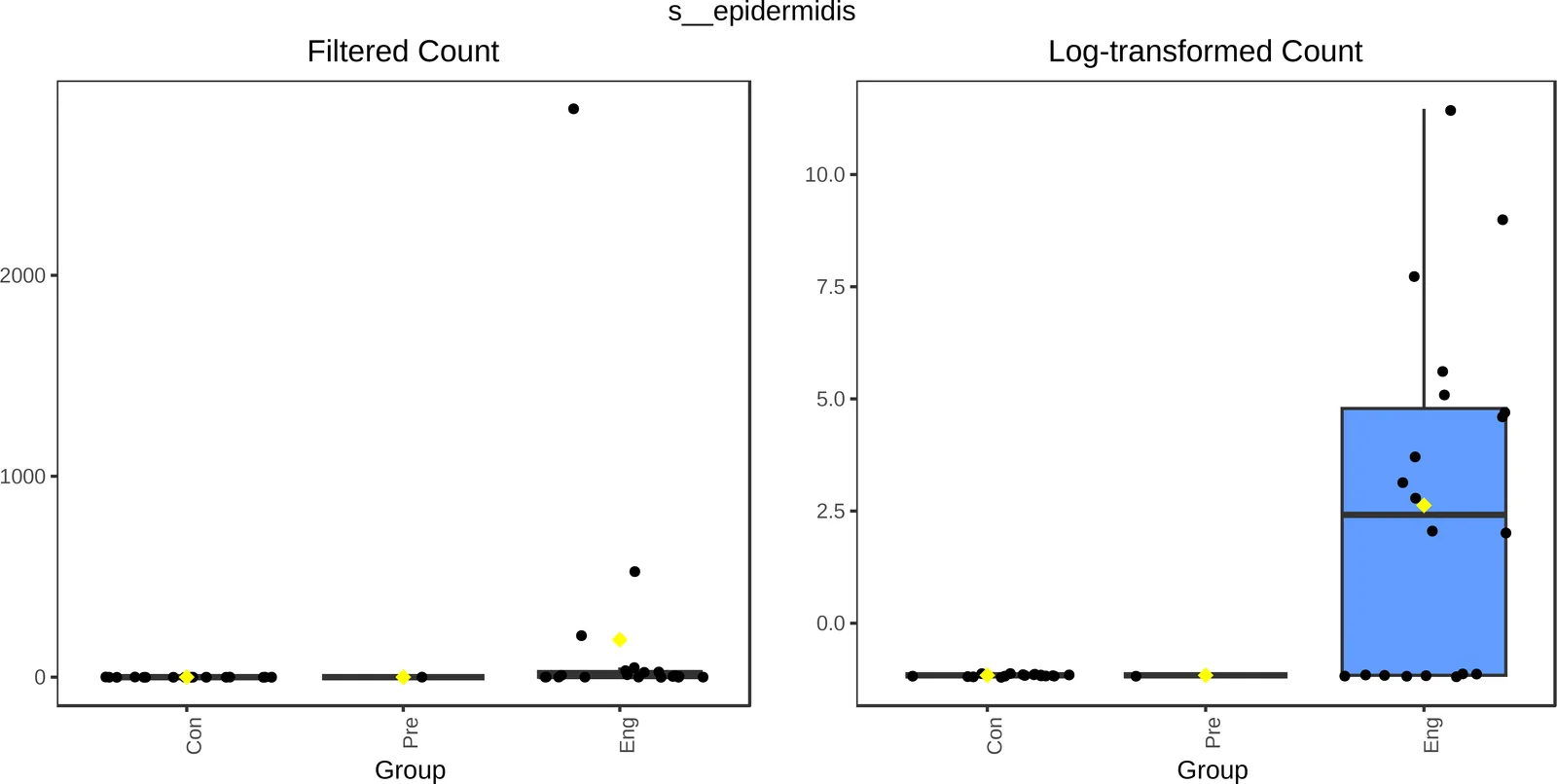
*Staphylococcus* abundance differences between pre-conditioning, time of engraftment, and control groups. Filtered counts refer to 16S rRNA amplicon sequence read counts for each group. Log-transformed values of the 16S rRNA sequence read counts show more obvious differences determined by DESeq2 analysis at FDR < 0.1.

A comparison on the genus level revealed *Staphylococcus* at higher levels post-transplant at the time of engraftment vs pre-conditioning (Fig. 4). This was one of the few taxa that was at higher relative levels in allo-HSCT patients at the time of engraftment vs that in healthy controls.

**Fig 5:**
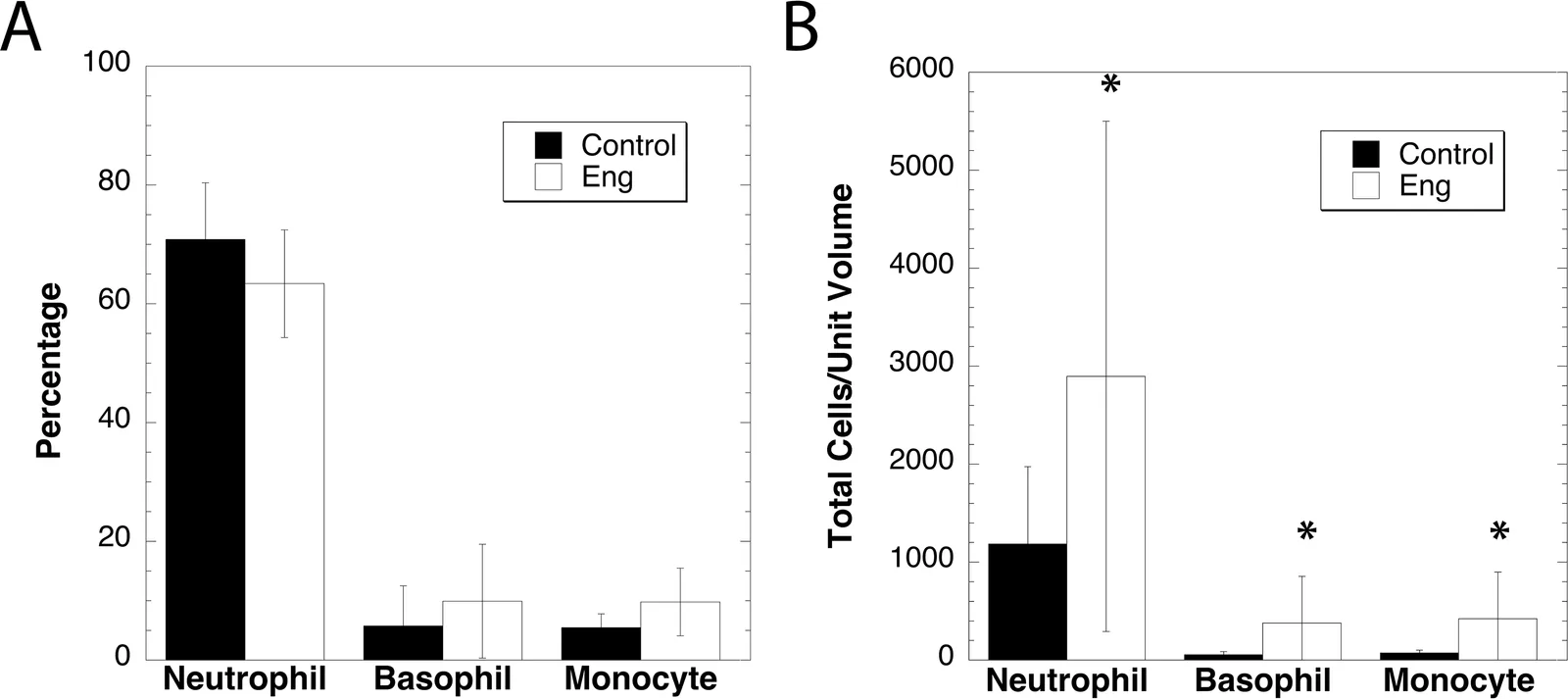
Salivary innate immune profiles, showing cell type percentages and cell concentrations per unit volume of saliva of healthy control vs that of HSCT patients at the time of engraftment. Panel **A** compares percentage cell type vs total cells in the two groups, differences compared using Mann-Whitney test. (**B**) Similar test as for panel **A** except total cells per unit volume of saliva. * indicates values significantly different for neutrophils, FDR = 0.041; basophils, FDR = 0.0066; and monocytes, FDR = 0.0055.

To investigate the relationship between innate immune cells in the saliva of post-transplant vs control patients, we compared the percentage of neutrophils, basophils, and monocytes out of total cells in saliva for the control and post-HSCT groups (Fig. 5A). Since normality cannot be assumed for these samples, the non-parametric Mann-Whitney *U* test was used. The average percentage of neutrophils among all cells in the post-transplant saliva at the time of engraftment trended toward being less than that in control patients (post-transplant, 63.4 ± 9.1; control, 70.8 ± 9.5, *P* = 0.052, FDR = 0.153). When comparing basophil and monocyte percentages between groups, we observed no significant differences in the composition, with FDR = 0.42 and 0.163, respectively. Alternatively, we considered the level of innate immune cells per unit volume of saliva sample for each group as another measure of immune response (Fig. 5B). Most significantly, monocytes per unit volume of sample were at a high level in patients at the time of engraftment in comparison to control patients. Similarly, basophils and neutrophils per unit volume of sample were much higher in patients at the time of engraftment than controls. Total saliva volume collected over 5 min was 5.5 ± 4.0 mL in patients at the time of engraftment and 7.7 ± 4.0 mL in healthy control (*P* < 0.245).

Given the irregularities in the granulocyte levels at the time of engraftment vs controls, we sought to determine if there was a relationship between oral microbiome alpha diversity among different participants and the different values of salivary granulocytes. This relationship was examined using Spearman correlation, and the results are tabulated in Table 2 below. Differences were noted in percentage neutrophils and total neutrophils/unit volume vs alpha diversity, which trended as being significant. Given the variation in saliva volume between donors, a third measurement was total salivary neutrophils collected. Similar relationships were explored for both basophils and monocytes, but no statistically significant associations were observed (Table S1).

**TABLE 2:** Correlation between alpha diversity of the microbiome of saliva and neutrophil levels in saliva at the time of engraftment^*a*^

Saliva microbiome/ saliva cells	Chao1 index (*ρ*, *P*, FDR)	Shannon index (*ρ*, *P*, FDR)
Neutrophil percentage	0.601, *P* = 0.039, FDR = 0.078	0.336, *P* = 0.286, FDR = 0.286
Total neutrophil/unit volume	0.629, *P* = 0.028, FDR = 0.056	0.399, *P* = 0.199, FDR = 0.286
Total neutrophil collected	0.538, *P* = 0.071, FDR = 0.142	0.399, *P* = 0.199, FDR = 0.286

^*a*^ Neutrophil percentage of all intact cells. Total neutrophils per unit volume of saliva. Total neutrophil collected (total neutrophil × saliva volume collected in 3 min). FDR correction was applied for testing multiple neutrophil metrics.

### Association of saliva microbiome with blood innate immune cells

Because the relationships between oral neutrophil measures and alpha diversity at the time of engraftment showed a trend toward significance, we next examined whether there were relationships between blood cell parameters and alpha diversity. Using Spearman correlation, several positive associations were found, and the results are tabulated in Table 3 below. In addition to a strong association with absolute neutrophil count, Shannon diversity indices indicated a positive correlation with hemoglobin and trended as such with white blood cell count and hematocrit.

**TABLE 3:** Spearman rank correlation between alpha diversity of microbiome of saliva and inflammation-related cells and red blood cell measures derived from the CBC^*a*^

Saliva microbiome/ blood parameter	Chao1 index (*ρ*, *P*, FDR)	Shannon index (*ρ*, *P*, FDR)
Absolute neutrophil count	0.481, *P* = 0.0274, FDR = 0.137	0.526, *P* = 0.0142, FDR = 0.0355
White blood cells	0.418, *P* = 0.0592, FDR = 0.148	0.450, *P* = 0.0408, FDR = 0.051
Hematocrit	0.304, *P* = 0.179, FDR = 0.224	0.463, *P* = 0.0348, FDR = 0.051
Platelet count	0.274, *P* = 0.236, FDR = 0.236	0.372, *P* = 0.0964, FDR = 0.0964
Hemoglobin	0.325, *P* = 0.149, FDR = 0.224	0.552, *P* = 0.0045, FDR = 0.0225

^*a*^ Hemoglobin, g/dL; hematocrit, percentage volume; all others are counts per unit volume. FDR correction was applied for testing multiple CBC parameters.

## DISCUSSION

This study explored the use of saliva analysis to better understand changes in the oral microbiome following allo-HSCT. Saliva was chosen as it is representative of most, if not all, oral surfaces, and its examination can be used to characterize both microbes and host cells present (22). As others have shown, there was a low level of alpha diversity even prior to conditioning for allo-HSCT, likely due to frequent antimicrobial usage, abnormal immune system, cancer treatment, or other factors (Fig. 1) (16). This was even lower at the time of engraftment when restoration of the innate immune system is well underway (23). Of course, this effect may be dependent on the treatments for the side effects of allo-HSCT (24). We noted that beta diversity measures similarly showed substantial differences in saliva between samples collected prior to allo-HSCT and at the time of engraftment, with both states differing from controls (Fig. 2).

It is difficult to compare differences in oral bacterial taxa observed in the current study vs earlier allo-HSCT longitudinal studies due to differences in types and times of sample collection. Heidrich et al. (9) observed differences in supragingival bacteria at the time of preconditioning and engraftment 14–30 days post-transplant in adults. A second study with allo-HSCT adult patients with saliva samples taken at the time of preconditioning and then 7–13 days later noted many differences in bacterial abundances (25). Among these two studies and the current study, a consistent observation was the increase in relative abundance on the genus level of *Staphylococcus* 20–30 days after allo-HSCT (Fig. 4).

A major component of recovery for allo-HSCT is the restoration of a normal innate immune system, which is defined as the end of hematological neutropenia and the restoration of normal or near-normal levels of blood neutrophils (24, 26). The assay for saliva innate immune cells relied on DNA methylation-based identification of cell types (27, 28). Surprisingly, measures of innate immune cells in the oral cavity revealed higher levels of neutrophils, basophils, and monocytes per unit volume of saliva in allo-HSCT patients at the time of engraftment vs age- and gender-matched controls (Fig. 5). One might speculate that this is due to a higher level of oral mucositis in these patients, but there is evidence that oral mucositis is associated with lower levels of neutrophils in allo-HSCT recipients (29). Typically, at this point, if oral mucositis was present in the patient, it is resolved or resolving (30). Unfortunately, the oral mucositis state of patients was not known for many of the patients. We note the control group, while similar in age and gender to the patient group, had many differences from the patient group, which could contribute to differences in oral immune cells and saliva. Specifically, for the saliva immune cell study, which had a more limited data set, the control group was older, 58.0 years vs 49.9 years for the allo-HSCT patients. This is unlikely to be a large factor in the result, as typically inflammatory cells increase, at least in blood, in older adults (31, 32). Allo-HSCT patients often receive antifungals, antivirals, and antibiotics; all patients undergo cytotoxic therapy as conditioning prior to the transplantation and typically have long-term blood disorders, which themselves or their treatments may contribute to differences in oral inflammatory cells. Or perhaps simply, these elevated levels of inflammatory cells could be an indication of a system that is coming to equilibrium after the shock of immune system ablation followed by infusion of immune cells.

In their comprehensive longitudinal study of oral microbiota changes after allo-HSCT, Heidrich et al. (17) noted a subset of patients who showed large changes in beta diversity (and typically alpha diversity) of their oral mucosal microbiome pretreatment vs at the time of engraftment. These patients trended as showing, by 30 days after engraftment, low levels of neutrophil and lymphocyte blood counts and long-term poor overall survival. This suggested poor recovery of the oral mucosal microbiome may be a cause or effect of poor restoration of normal blood cell count and immune system function after allo-HSCT. In this study, we observed patients with low alpha diversity based on the number of species at the time of engraftment also trended as showing lower levels of salivary neutrophils (Table 2). The absolute neutrophil count in the blood was similarly correlated with low levels of alpha diversity in the saliva or at least trended that way after correction for multiple testing (Table 3). Surprisingly, we also noted that the levels of blood platelets and hemoglobin were also directly related to the level of Shannon Diversity index at the time of engraftment or trended as such (Table 3). This finding is comparable to earlier observations in the gut, where higher microbiome alpha diversity at the time of engraftment was associated with higher lymphocytes 3 months later in a patient (33), suggesting the key role of the gut microbiome in reconstitution of the hematopoietic system after allo-HSCT. We also note that several groups have shown the efficacy of providing fecal bacterial transplants during allo-HSCT to increase the chance of successful outcomes (34–36). It is difficult to determine whether the status of the oral microbiome alone is sufficient to affect the systemic immune system or whether major sites of microbiome, such as the gut, mouth, and skin, each contribute to or respond to the arms of the immune system that are intimately involved with their specific sites. Others have speculated about this earlier (34), and there are indeed some suggestions that microbes at different body sites can be linked to different types of adverse outcomes, from allo-HSCT or other applications of cancer chemotherapy (37, 38). It is well known that the human microbiome plays a role in the original immune system’s development (39). In agreement with earlier work on the gut microbiome, we see the potential for the maintenance of the normal oral microbiome during engraftment contributing to the establishment of the new immune system.

A strength of this study is that full-length 16S rRNA amplicon sequencing allowed more certainty on taxa identification. The use of methylation analysis to identify oral cells demonstrated the potential of this assay. Limitations of this study are the small size, the lack of leukocyte data for pre-transplant samples, and the lack of oral mucositis data for all patients. However, the findings reinforce earlier results regarding oral microbiome at the time of allo-HSCT and at the engraftment point. It is reasonable to assume that, like in the blood, salivary immune cell depletion occurs with HSCT conditioning. If this is the case, this work also allows one to speculate about salivary innate immune cell restoration after HSCT at the time of engraftment and suggests a possible connection to oral microbiome state at that time.

## MATERIALS AND METHODS

### Study design and

A total of 21 individuals participated in this research study. Inclusion criteria were adults who received an allo-HSCT at Northwestern Memorial Hospital between July 2022 and June 2023 and agreed to be in the study—22 patients. Exclusion criteria included patients who did not provide a saliva sample at the time of transplant or at the time of engraftment—one patient. Control saliva donors did not undergo allo-HSCT, nor did they suffer from any of the ailments that require that treatment. Control group consisted of patients appearing for a check-up and staff at the University of Illinois Chicago Dental Clinic, matched by age and gender, from whom samples were collected in the morning or at midday in the same manner as that of the allo-HSCT patients.

### DNA extraction and 16S rRNA ampliconsequencing

Patient samples were derived from stimulated saliva accumulated from chewing paraffin wax over 5 min and processed as described earlier (40). Collected saliva samples were stored on ice for up to 1 h prior to the addition of equal volume of DNA/RNA Shield (Zymo Research), vortexed, and stored at −80°C. DNA was extracted from saliva in preservative using the Quick-DNA Fungal/Bacteria Miniprep Kit (Zymo Research, Irvine, CA, USA). DNA samples were processed for PCR amplification and sequencing, targeting near-full-length microbial 16S rRNA genes using a two-stage PCR protocol for PacBio, in a protocol similar to that used for Illumina sequencing (41, 42). Sequencing was performed on a PacBio Revio instrument employing Kinnex chemistry. Library preparation and sequencing were performed under the direction of the Genomics and Microbiome Core Facility at Rush University. Negative controls were samples that started with H_2_O instead of saliva DNA.

### Host cell measurement in saliva

Half of 1 mL saliva and 0.5 mL DNA RNA shield were combined and stored at −80°C. They were submitted to Precision for Medicine (Berlin, Germany/Bethesda, MD, USA) for Epiontis ID profiling of innate immune cells and total cells by bisulfite-based conversion of unmethylated cytosine to uracil, while methylated cytosine is unaltered. Marker genes with methylation differences that are specific to one cell type allow counting of that cell via RT-PCR targeted to those specific sites (27, 28). This allows measurement of various immune cells without the use of flow cytometry or microscopy. Total cells were measured by RT-PCR amplification of a section of the GAPDH gene not subject to methylation.

### Bioinformatics and statistics

PacBio 16S rRNA amplicon sequencing of the 16S rRNA gene yielded an average of 33,525 reads per sample after demultiplexing, using 30% of the sequencing run. Minimum read totals for a sample were 21,204 and maximum 56,350. Single-end, quality-filtered reads with lengths between 1,000and 1,600 bp were subjected to denoising and amplicon sequence variant (ASV) identification using DADA2, with PacBio default parameters implemented in QIIME2 (max_mismatch: 2, indels: false, trunc-len: 0, and chimera removal: consensus) (43–45). Alignment to the HOMD microbiome database (46) used BLAST.

For statistical analysis of the study population demographics and clinical factors, descriptive statistics were used to summarize the characteristics of patients. Categorical variables were summarized using frequencies and percentages. Relationships between categorical variables were tested with the two-tailed Fisher’s exact test. Continuous variables were presented as mean and standard deviation. Spearman correlation coefficients were estimated to measure the relationship between two continuous variables. To assess the correlations between categorical clinical factors and numerical variables, *t*-tests were conducted when data followed a normal distribution; otherwise, the Mann-Whitney test was used instead (47). Differences in specific microbiota taxonomic abundances between groups were tested using DESeq2 based on the negative binomial distribution. This was performed after setting abundance values of ≤2 reads to zero (baseline) in the abundance table and eliminating taxa that appeared in 10% or fewer participants. Taxa differentially abundant at FDR < 0.1 or lower are presented for these large data sets. For small-scale multiple testing, significance was set at FDR < 0.05. ASVs not assigned to the HOMD 16S rRNA gene database were eliminated prior to statistical analysis as they were grouped together in the output abundance table.

## Data Availability

Sequence data in the form of 16S rRNA fastq files are available through the Sequence Read Archive with primary accession code PRJNA1394480.

## SUPPLEMENTAL MATERIAL

Tables S1 and S2 spectrum.00118-26-s0001.docx Table S1: More blood parameters. Table S2: Control vs time of engraftment saliva microbiome.

ASM does not own the copyrights to Supplemental Material that may be linked to, or accessed through, an article. The authors have granted ASM a non-exclusive, world-wide license to publish the Supplemental Material files. Please contact the corresponding author directly for reuse.
