# Supplementary material for: State of oral innate immunity and salivary microbiome with allogeneic hematopoietic stem cell transplant
Source: Microbiol Spectr. 2026 Aug 10;14(9):e00118-26. doi: 10.1128/spectrum.00118-26 (PMC13532226; doi:10.1128/spectrum.00118-26)
Supplement: Supplementary file 1 [file spectrum.00118-26-s0001.docx]

**Supplemental Data:**

**Oral innate immune and salivary microbiome state with allogenic hematopoietic stem cell transplant**

**Adami, G.R.^1,#^, Fine, M.^1,#^, Schwartz J.L.^1^, Wotjowicz P^2^, Moreira J.^3^**

**Table S1.** Correlation between alpha diversity of oral microbiome and basophil and monocyte levels in saliva at time of engraftment.

| **Blood Parameter** | **Chao1 Index (ρ, p)** | **Simpson Index (ρ, p)** | **Shannon Index (ρ, p)** |
| --- | --- | --- | --- |
| Basophil % | -0.073, p = 0.832 | 0.491, p = 0.125 | 0.491, p = 0.125 |
| Basophils/unit volume | 0.018, p = 0.958 | -0.155, p = 0.650 | -0.109, p = 0.750 |
| Total Basophils | 0.036, p = 0.916 | 0.027, p = 0.937 | -0.036, p = 0.916 |
| Monocyte % | -0.182, p = 0.593 | -0.318, p = 0.340 | -0.282, p = 0.401 |
| Monocytes/unit volume | 0.073, p = 0.832 | -0.191, p = 0.574 | -0.127, p = 0.709 |
| Total Monocytes | 0.064, p = 0.853 | -0.082, p = 0.811 | -0.118, p = 0.729 |

**Table S2 Differentially abundant species in saliva of healthy controls (Con) vs. that from HSCT patients at time of engraftment (Eng) as identified using DESeq2 at FDR < 0.1**

| **Taxon** | **log2FoldChange** | **lfcSE** | **Pvalues** | **FDR** |
| --- | --- | --- | --- | --- |
| *Streptococcus cristatus* | 9.1079 | 0.89813 | 3.63E-24 | 6.25E-22 |
| *Aggregatibacter segnis* | 8.8529 | 0.93447 | 2.70E-21 | 2.32E-19 |
| *Neisseria elongata* | 8.6304 | 0.98704 | 2.26E-18 | 1.29E-16 |
| *Haemophilus sputorum* | 9.6617 | 1.1558 | 6.30E-17 | 2.46E-15 |
| *Gemella morbillorum* | 7.1117 | 0.85229 | 7.16E-17 | 2.46E-15 |
| *Capnocytophaga haemolyticus* | 9.0966 | 1.2314 | 1.50E-13 | 4.29E-12 |
| *Alloprevotella sp__HMT_914* | 8.5535 | 1.2664 | 1.44E-11 | 3.53E-10 |
| *Lautropia mirabilis* | 6.6566 | 1.0098 | 4.34E-11 | 9.32E-10 |
| *Leptotrichia sp__HMT_417* | 7.9908 | 1.2776 | 3.99E-10 | 7.63E-09 |
| *Hallella sp__HMT_351* | 6.1288 | 1.0747 | 1.18E-08 | 2.03E-07 |
| *Granulicatella elegans* | 6.956 | 1.2428 | 2.18E-08 | 3.41E-07 |
| *Cardiobacterium hominis* | 5.6733 | 1.0748 | 1.30E-07 | 1.87E-06 |
| *Tannerella serpentiformis* | 6.1609 | 1.2607 | 1.02E-06 | 1.36E-05 |
| *Lachnospiraceae [G2] bacterium_HMT_096* | 6.8967 | 1.4726 | 2.82E-06 | 3.47E-05 |
| *Capnocytophaga leadbetteri* | 6.6033 | 1.4907 | 9.44E-06 | 0.00010826 |
| *Prevotella intermedius* | 6.7429 | 1.5419 | 1.23E-05 | 0.00013168 |
| *Veillonella parvula* | -2.9249 | 0.67763 | 1.59E-05 | 0.00016044 |
| *Lacticaseibacillus;rhamnosus* | -7.0166 | 1.6575 | 2.30E-05 | 0.00021998 |
| *Staphylococcus epidermidis* | -6.3282 | 1.5474 | 4.32E-05 | 0.00039147 |
| *Absconditicoccaceae [G1] bacterium_HMT_875* | 7.0126 | 1.7207 | 4.59E-05 | 0.00039492 |
| *Eikenella corrodens* | 5.8927 | 1.4567 | 5.23E-05 | 0.00042807 |
| *Streptococcus salivarius* | 3.3845 | 0.84393 | 6.06E-05 | 0.00047391 |
| *Capnocytophaga endodontalis* | 6.3187 | 1.5812 | 6.43E-05 | 0.00048122 |
| *Haemophilus parahaemolyticus* | 7.1399 | 1.8218 | 8.89E-05 | 0.00063682 |
| *Fuosbacterium periodonticum* | 7.3578 | 1.894 | 0.00010239 | 0.00070442 |
| *Hoylesella koreensis* | 5.546 | 1.738 | 0.0014175 | 0.0093773 |
| *Leptotrichia shahii* | 6.375 | 2.0083 | 0.001502 | 0.0095681 |
| *Alloprevatella rava* | 5.2449 | 1.6663 | 0.0016457 | 0.010109 |
| *Parvisynbacter sp__HMT_348* | 5.1561 | 1.6937 | 0.0023323 | 0.013685 |
| *Aggregatibacter kilianii* | 6.714 | 2.2105 | 0.002387 | 0.013685 |
| *Corynebactyerium matruchotii* | 5.4201 | 1.8341 | 0.0031245 | 0.017336 |
| *Segatella sp__HMT_305* | 5.2707 | 1.8056 | 0.0035114 | 0.018874 |
| *Nanosynbacter sp__HMT_352* | 3.2858 | 1.1328 | 0.003724 | 0.01941 |
| *Anaerovoracaceae [G1] sulci* | 3.343 | 1.1639 | 0.0040751 | 0.020615 |
| *Oribacterium sp__HMT_078* | -5.9634 | 2.0975 | 0.0044671 | 0.021953 |
| *Hoylesella loescheii* | -3.4398 | 1.2359 | 0.0053835 | 0.025721 |
| *Actinomyces naeslundii* | -4.8138 | 1.7818 | 0.0069006 | 0.032078 |
| *Streptococcus parasanguinis* | -2.4373 | 0.95922 | 0.011058 | 0.050051 |
| *Porphyromonas sp__HMT_930* | 5.1866 | 2.0525 | 0.011504 | 0.050738 |
| *Neisseria subflava* | 3.6354 | 1.4823 | 0.014186 | 0.061001 |
| *Kingella denitrificans* | 4.9029 | 2.092 | 0.019099 | 0.080122 |
| *Burkholderia cepacia* | -2.6531 | 1.1438 | 0.02036 | 0.08338 |
| *Campylobacter concisus* | -1.34 | 0.58791 | 0.02265 | 0.089236 |
| *Streptococcus mutans* | 4.3748 | 1.9281 | 0.023273 | 0.089236 |
| *Parvimonas sp__HMT_110* | 3.1018 | 1.3678 | 0.023347 | 0.089236 |
| *Hoylesella bacterium_HMT_075* | 2.7872 | 1.2426 | 0.024897 | 0.093093 |
| *Haemophilus sp__HMT_036* | 5.1017 | 2.2921 | 0.026034 | 0.095273 |
| *Selenomonas sputigena* | 2.2625 | 1.0285 | 0.02781 | 0.099652 |
| *Streptococcus salivarium* | -5.1808 | 2.373 | 0.029021 | 0.10187 |
